# Yes, research can inform health policy; but can we bridge the 'Do-Knowing It's Been Done' gap?

**DOI:** 10.1186/1478-4505-9-23

**Published:** 2011-06-16

**Authors:** Stephen R Hanney, Miguel A González-Block

**Affiliations:** 1 Health Economics Research Group, Brunel University, Uxbridge, UK; 2 Center for Health Systems Research, National Institute of Public Health, Cuernavaca, Mexico

## The impact of health research: From 'Know-Do' to 'Do-Knowing It's Been Done'

*Health Research Policy and Systems (HARPS) *is publishing a supplement of papers that is well timed as it includes accounts of various strategies used by research organisations to strengthen the research to policy and practice interface [1]. It comes as the World Health Organization (WHO) plans for the 2012 edition of its flagship publication, the World Health Report, which will focus on the role of research in improving the health status of populations [2].

The forthcoming World Health Report, to be entitled: 'No Health Without Research', reflects an ever-growing focus on the vital role of health research, and how best to bridge the 'Know-Do' gap. In 1990 the independent Commission on Health Research for Development published a landmark report, *Health Research: Essential Link to Equity in Development *[3]. The WHO has been playing an increasingly important part in promoting the role of health research. It organised the Mexico ministerial summit on health research [4] and the accompanying *World Report on Knowledge for Better Health: Strengthening Health Systems *[5]. That was followed by the second ministerial summit at Bamako [6] and the First Global Symposium on Health Systems Research organised by the WHO/Alliance for Health Policy and Systems Research at Montreux in November 2010.

The specific role of health research in informing health policies has always been a major part of the analysis about the importance of health research [7]. In 2003 *HARPS *published a review and analysis of the topic [8] that had been undertaken as part of the lead-up to the Mexico summit. That paper made an early claim that, 'A full review of the many possible meanings of research impact reveals that there may be more utilisation in policymaking than is sometimes recognised.'[8]

The various overlapping themes in the literature include:

1. promoting the greater use of research and identifying the facilitators of and barriers to research making an impact on policy, which is sometimes framed as part of the debate about how best to bridge the 'Know-Do' gap;

2. describing specific attempts to enhance the impact made by research on policy;

3. one-off explorations of how far research has informed health policies in specific cases; and

4. developing systematic methods to assess and monitor the impact made by health research on policies, which could seen as addressing what we are calling the 'Do-Knowing It's Been Done' gap.

Various studies address these themes, often in overlapping ways. Whilst interest and activities have been intensifying in the last decade, it is important to recognise there have been major long-standing attempts within some health research systems to develop approaches in which policymakers and researchers work together to identify priorities for research that will meet the needs of policymakers. A large-scale formative evaluation by Maurice Kogan and Mary Henkel of one attempt in the English health department's R&D system was published as early as 1983 [9]. It highlighted many of the difficulties in getting policymakers and researchers to develop the productive long-term relationships to improve the impact. It also, however, developed many of the concepts that are now used more widely, such as the importance of the collaborative approach, the role of knowledge brokers and the role of receptor bodies. A second edition [10] highlighted the continuing attempts to address the issue in the English health research system, and a subsequent paper in *HARPS *provided a full account of how 30 years of reform has resulted in a health research system that has had successes in meeting the needs of various stakeholders, including some policymakers [11].

## The recent contribution made by *HARPS *to the evolving analysis and debates

In *HARPS *we have attempted to contribute to all of the four themes outlined above by publishing a range of relevant papers, series and supplements. In 2009 we published a supplement called *SUPPORT Tools for evidence-informed health Policymaking (STP) *which consisted of a series of guides on how to increase the impact of research on policy with an introduction by John Lavis, Andy Oxman and colleagues [12]. This is a major attempt to reduce the 'Know-Do' gap and, despite the various examples of research making an impact, there is clearly much work still to do to reduce that gap. In turn, that series helped inform an innovative approach from Melissa Pearson and colleagues whose article in *HARPS *combines policy sciences traditions with the focus on pathways provided by the SUPPORT tools to promote evidence informed policymaking. These combine to facilitate prospective policy analysis that informed policymaking on intentional self poisoning in Sri Lanka [13].

In May 2010 a symposium was held at Harvard University, Boston, USA, to mark the 20^th ^anniversary of the report from the Commission on Health Research for Development. Julio Frenk and Lincoln Chen wrote a Commentary on the symposium that was published in *HARPS*. Frenk and Chen observe that the participants 'underscored the imperative that knowledge be translated into evidence that can guide policy and implementation.' [14]. At a more specific level a paper in *HARPS *by Rajeev Gupta and colleagues [15] highlights the body of evidence that should be translated into policy for cardiovascular disease control in India.

The interest in many countries in the field of increasing research use is illustrated by a range of papers published in *HARPS *in the last 16 months. One paper considers the role being played by the print media in 44 countries in Africa, the Americas, Asia, and the Eastern Mediterranean as one dimension of the climate for evidence-informed health systems [16]. Two linked papers describe surveys used to gather opinions about bridging the gaps between research, policy and practice in 10 low and middle income countries [17,18]. Various initiatives in this field from The Netherlands have also been reported, including an exploration of barriers between epidemiological research and local health policy formation [19] and an approach for assessing the use (including impact on policy) of research produced by one of the Dutch university medical centres [20]. A further paper describes a framework for developing an evidence-based comprehensive tobacco control program in Israel [21].

A key paper in *HARPS *by Godfrey Woelk and colleagues from December 2009 won that year's prize in the Medicine category for the best groundbreaking research published in any of BioMed Central's journals: *Translating research into policy: lessons learned from eclampsia treatment and malaria control in three southern African countries *[22]. This success is another indication of the increasing importance of the topic, and the paper provides further important examples of the use of research in health policy and an insightful analysis of barriers and facilitators.

As noted, there have been various other one-off, and/or small scale, studies on the impact of research on policy; some indicating a high level of influence. These include studies of health technology assessments in Quebec, Canada [23] and analysis of 44 operations research projects aiming to improve reproductive health services in Guatemala [24].

## It's time to recognise the increasingly important role played by health research

Building on our own paper in 2003 [8], as editors of *HARPS *we have been pushing the case for increasing recognition that health research does impact on policy more frequently than is often acknowledged [25]. Of course not all heath research can make an impact on policy, nor should this be expected. However, whilst it is important that a major focus is maintained on bridging the 'Know-Do' gap, it is also time that more attention was paid to the 'Do-Knowing It's Been Done' gap so as to ensure that evidence is captured about when and how health research makes an impact on policy.

Developing robust techniques to assess the impact of health research has been recognised in the 1990s as being important for various reasons: it provides accountability for funds spent, justification for future spending and also helps identify ways to organise health research so as to achieve greater impacts in the future [26]. If real progress is to be made in evaluating the different mechanisms used to increase the use of research, it could be argued that it is important to know what impact has been made by the research that is translated into action. As a corollary to the collaborative approach developed by Kogan and Henkel [9], there is recognition of the need to focus on issues at the interfaces between policymakers and researchers as a way of helping to understand how the impact on policy has come about [26,27]. The Payback Framework developed in the mid 1990s by Buxton and Hanney [26], and elaborated in an article in *HARPS *[27], addresses these concerns. It incorporates consideration of the permeability of the interfaces between the research system and the wider political and healthcare systems. The issue of permeability at the interfaces includes questions about how far the wider healthcare, political and social systems can collaborate with researchers to produce an agenda that will engage researchers, and how far the findings from research can make an impact on the wider systems. The Payback Framework consists not only a multi-dimensional categorisation of benefits from research, but also a model of the processes of research production and use that can help in assessing the benefits achieved [26]. In this approach, therefore, the analysis of the value of the interface mechanisms used to help achieve impacts is informed by the assessment of the actual impacts that arise from the translation of the research.

Such considerations could be important in progressing effective implementation of the framework developed by Lavis and colleagues [28] for evaluating what has been done to promote efforts to link research to action. Their framework covers a wide range of mechanisms that might be used, including: push efforts by producers of research; user pull efforts; exchange efforts involving researchers and users working together in ways such as through the use of knowledge brokers; and integrated efforts. Their framework also recognises the importance of evaluating such mechanisms. Approaches such as the Payback Framework [26] provide a way to assess the wider impacts of the research that is translated through the various possible translation mechanisms available. Therefore, these ways of assessing the wider impacts might assist attempts to evaluate the effectiveness of the various translation mechanisms.

Whereas some attempts to assess the impact of research look just at policy, other frameworks, such as the Payback Framework, include impact on policy as part of a multi-dimensional categorisation of benefits that also includes health and health equity benefits as well as broader economic benefits [26]. Indeed, establishing the impact on policy (especially using a broad definition to include clinical policies) can be seen as a key factor in helping to identify the wider impacts [29].

To demonstrate when and how research has an impact on policy, studies can either start with research and trace the impact forwards, or start with policies and attempt to trace the impact backwards to the relevant research that might have influenced the policy. In our 2003 review we suggested that the evidence indicated it could be less difficult to trace the impact forwards than it was to work backwards, and this opinion was strengthened by a review in 2007 [29]. Whilst it might be more difficult to trace policy impacts back to specific pieces of research, there is increasing evidence of policymakers acknowledging research can inform their decision-making. In a recent study of national policymakers in six countries Adnan Hyder and colleagues show that whilst there are various barriers to the use of research, the policymakers interviewed, 'were unequivocal in their support for health research and the high value they attribute to it'[30].

Several issues will have to be addressed in any attempts to put greater emphasis on bridging the 'Do-Knowing It's Been Done' gap. There are the different, although related, processes of using specific research results through commissioning or pushing primary research, and using secondary research through reviews and synthesis. Indeed, there is sometimes a lack of clarity in the literature about whether the emphasis is on enhancing (and assessing) the use of the findings produced by researchers within the local healthcare/research system, or on enhancing (and assessing) the use of the relevant parts of the global body of health research. Clearly both activities are important, and there is an increasingly diverse range of approaches used for pulling together locally generated and synthesised global knowledge in a way that is most appropriate for policymakers in specific countries. Furthermore, there are overlaps in that a collaborative approach might be as valuable in getting policymakers to pay attention to secondary research as it is with primary research. However, if the case is to made for funding local primary research in low and middle income countries (because, for example, its findings are more likely to be relevant to policymakers in those countries), then it is important that sufficient attention is given to assessing the impact of such research on policy.

## Extending the analysis: a new supplement in *HARPS*

*HARPS *is now publishing a supplement consisting of a diverse range of papers first presented at a conference on getting research into policy and practice in the field of sexual and reproductive health (SRH) and HIV/AIDS. These papers cover a wide range of topics, many of which are related to the main themes identified above, including the importance of focussing on ways not only of enhancing the impact of research (on policy and practice) but also of demonstrating that impact has been achieved. In an introductory paper Sally Theobald and colleagues state:

The contributors to this supplement provide a body of critical analysis of communications and engagement strategies across the spectrum of SRH and HIV/AIDS research through the testing of different models for the research-to-policy interface. They provide new insights on how researchers and communication specialists can respond to changing policy climates to create windows of opportunity for influence [1].

Here we present a flavour of the wide range of approaches and topics described by giving a brief outline of key points from three contrasting papers. Eleanor Hutchinson and colleagues examine national policymaking for cotrimoxazole as a preventive therapy for HIV infected individuals in Malawi, Uganda and Zambia [31]. The approach adopted by the authors was informed by a recent overview of the health policy literature in low and middle income countries. That review concluded with a call for analyses which consist of comparative, multi-country studies using rigorous case studies which deliberately seek to explain health policy changes in these settings [32]. Hutchinson and colleagues identify several factors that influenced the variable impact of the research in the different countries, and observe that while the findings from randomised controlled trials were not necessarily translated into policy so swiftly, 'local operational research results seem to have been taken up more quickly'[31].

Rose Oronje and colleagues [33] use a case study approach to describe the experiences of the African Population and Health Research Center in Nairobi, Kenya, and its partners, in cultivating the interest and building the capacity of the media in evidence-based reporting of reproductive health issues in sub-Saharan Africa. They conclude that the media can play a valuable role in communicating important research findings and raising the profile of overlooked and contentious public health issues to the public, including political leaders, policymakers and key stakeholders [33].

Alan Whiteside and Fiona Henry [34] examine how, where and why there was a considerable impact made by the 2007 report on the HIV and AIDS epidemic in Swaziland entitled *Reviewing 'Emergencies' for Swaziland: Shifting the Paradigm in a New Era *[35]. Adopting the approach of tracing the impact forwards from the original research, as described above, they explore how following a targeted communications effort, that report succeeded in raising the profile of the epidemic as a humanitarian emergency requiring urgent action from international organisations, donors, and governments. In the literature on assessing the impacts made by research on health policies it has been stressed that the quality of the research can be seen as an important factor in achieving impacts [8,26], and this is well-illustrated in Whitehead and Henry's conclusion that 'The credibility of both evidence and researcher play an important role in the use of research.'[34]. Finally, the authors end with a key observation that not only did the original report achieve many of its goals and spur an international dialogue around the issues, but also the evaluation of the report's impact provides, 'additional lessons, which can be applied to help maximise the impact of research in the future.'[34].

So, the new supplement in *HARPS *makes significant additions to the growing body of literature, from *HARPS *and elsewhere, that research can inform health policies, that there are various barriers and facilitators that should be analysed, and that it is also important to expand the analysis and bridge the 'Do-Knowing It's Been Done' gap.

## Competing interests

SH and MG-B receive funding from a range of organisations with an interest in health research, including some specifically mentioned in this editorial such as the World Health Organization and the Alliance for Health Policy and Systems Research.
